# Role of dendritic cells in the induction of regulatory T cells

**DOI:** 10.1186/2045-3701-1-20

**Published:** 2011-05-24

**Authors:** Rahul Kushwah, Jim Hu

**Affiliations:** 1 Physiology and Experimental Medicine Research Program, Hospital for Sick Children, 555 University Avenue, Toronto, Ontario, M5G 1X8, Canada; 2 Department of Laboratory Medicine and Pathobiology, University of Toronto, Toronto, Ontario, M5S 1A1, Canada

## Abstract

Dendritic cells (DCs) play a key role in initiating immune responses and maintaining immune tolerance. In addition to playing a role in thymic selection, DCs play an active role in tolerance under steady state conditions through several mechanisms which are dependent on IL-10, TGF-β, retinoic acid, indoleamine-2,3,-dioxygenase along with vitamin D. Several of these mechanisms are employed by DCs in induction of regulatory T cells which are comprised of Tr1 regulatory T cells, natural and inducible foxp3^+ ^regulatory T cells, Th3 regulatory T cells and double negative regulatory T cells. It appears that certain DC subsets are highly specialized in inducing regulatory T cell differentiation and in some tissues the local microenvironment plays a role in driving DCs towards a tolerogenic response. In this review we discuss the recent advances in our understanding of the mechanisms underlying DC driven regulatory T cell induction.

## Introduction

Dendritic cells (DCs) are professional antigen presenting cells and are essential mediators of immunity and tolerance. DCs are the key players in maintaining immune tolerance, for their ablation has been shown to result in autoimmunity, highlighting the active role that DCs play under steady state conditions in maintaining immune tolerance[1]. In order to prevent autoimmune reactions, self reactive lymphocytes need to be deleted or their function needs to be suppressed. The generation of normal lymphocyte repertoire which is largely self-tolerant depends on positive and negative selection, which occurs in the thymus and the process, is referred to as central tolerance. However, some self-reactive lymphocytes that escape thymic deletion enter peripheral tissues and the suppression of their function is needed to prevent autoimmune reactions, which is referred to as peripheral tolerance. Central tolerance in the thymus is largely mediated by cortical epithelial cells, medullary epithelial cells and thymic DCs and involves deletion of self reactive thymocytes along with induction of naturally occurring regulatory T cells (Tregs), which play a key role in maintaining self tolerance and suppressing a variety of pathological immune responses[2]. In contrast to central tolerance, peripheral tolerance is mediated by DCs through generation of Tregs and clonal deletion of self reactive T cells. Tregs generated in the periphery are thought to be important in controlling immune response to non-self antigens. Peripheral Tregs include IL-10 secreting Tr1 Tregs, inducible foxp3^+ ^Tregs, Th3 cells and double negative Tregs. DC induced generation of these Treg subsets is largely mediated by IL-27, TGF-β, IL-10, retinoic acid, indoleamine-2,3-dioxygenase and vitamin D. The generation of these Tregs is either mediated by tissue resident specific DC subsets with a specialized Treg inducing function or by the action of mediators present in local tissue microenvironment, which act on DCs and drive them to behave as tolerogenic DCs and induce Treg differentiation. In this review we provide an overview of the different mechanisms employed by DCs in generation of Tregs.

## Type 1 regulatory cells

**Figure 1:**
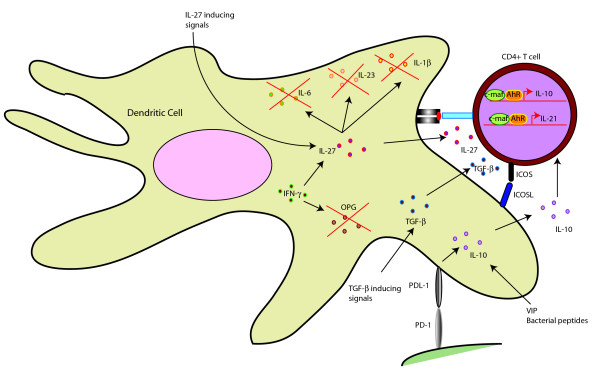
**DCs drive differentiation of Tr1 regulatory T cells**. DCs secrete IL-27, IL-10 and TGF-β1, which induce AhR and c-maf in T cells. AhR and c-maf physically associate with each other and activate IL-10 and IL-21 promoters, driving Tr1 differentiation. IL-27 suppresses production of Th17 inducing cytokines such as IL-1β, IL-6 and IL-23 and drives Tr1 differentiation. IFN-γ suppresses Th17 inducing osteoprotegerin (OPG) and drives IL-27 production, thereby promoting Tr1 differentiation. Furthermore, PD-1/PDL-1 signaling and bacterial peptides along with vasoactive intestinal peptide (VIP) drive IL-10 production which also induces Tr1 differentiation. Moreover, ICOS/ICOSL signalling as well as TGF-β production by DCs has also been implicated in driving Tr1 differentiation.

Type 1 regulatory T cells (Tr1) cells are a group of Tregs characterized by production of IL-10. Although initial studies pointed towards a central role of IL-10 in mediating Tr1 generation, recent studies indicate that Tr1 generation could also be dependent on IL-27. Both IL-10 and IL-27 are produced by DCs. Aryl hydrocarbon receptor (AhR), which is a ligand-activated transcription factor belonging to the basic helix-loop-helix-PER-ARNT-SIM family, is induced in Tr1 cells and during Tr1 differentiation, physically associates with c-maf, a transcription factor belonging to the family of basic region leucine zipper domain transcription factors and activates IL-10 and IL-21 promoters[3,4]. Studies to date have pointed towards a role of DC derived IL-27, IL-10, TGF-β1 along with a role of ICOSL signalling by DCs in induction of Tr1 cells. Figure 1 provides an overview of the pathways involved in DC mediated Tr1 differentiation.

### IL-27 production by DCs drives Tr1 differentiation

DCs cultured with Foxp3^+ ^Tregs secrete elevated levels of IL-10, IL-27 and TGF-β1, among which TGF- β1 and IL-27 are important for driving differentiation of Tr1 cells[5]. IL-27 suppresses production of Th17 polarizing cytokines IL-1β, IL-6 and IL-23 from DCs and acts on naive T cells to drive expression of the transcription factor c-maf, IL-21 and ICOS, which collectively drive differentiation of Tr1 cells[6,7]. Furthermore, IL-27 production by DCs also drives IL-10 transcription in T cells by activation of STAT1 and STAT3, which are recruited to the IL-10 promoter, further promoting differentiation of Tr1 cells [8]. Recently, IFN-γ has also been identified to promote DC induced Tr1 generation. Studies have shown that IFN-γ inhibits Th17 inducing osteoprotegerin (OPG) in DCs and instead promotes IL-27, which drives induction of Tr1 cells[7,9]. Hepatic DCs preferentially secrete IL-27 instead of IL-12 upon LPS stimulation, indicating that hepatic DCs may also act as inducers of Tr1 cells[10].

### IL-10 production by DCs drives Tr1 differentiation

Differentiation of DCs from bone marrow in the presence of IL-10 leads to generation of a CD11c^low^CD45RB^high ^DC subset with a plasmacytoid morphology and an immature phenotype[11]. These DCs secrete high amounts of IL-10 upon stimulation and drive differentiation of naive T cells into IL-10 secreting Tr1 cells. Similarly, skin derived langerhans cells have also been shown to produce IL-10 which can also contribute towards generation of Tr1 cells[12]. Similar to murine Tr1 cells, DCs secreting IL-10 also drives differentiation of human Tr1 cells, which is dependent on human leukocyte antigen (HLA)-G and (Ig like transcript) ILT4 molecules[13]. HLA-G, which is a non classical MHC I molecule, plays a central role in maintaining fetal-maternal tolerance during pregnancy and is expressed on IL-10 producing tolerogenic human DCs[14]. HLA-G binds to inhibitory immunoglobulin like transcript (ILT)-2 and ILT-4 receptors which has been shown to suppress DC maturation[15,16]. IL-10 produced by DCs acts in a positive feedback manner, sustaining HLA-G and ILT4 expression on DCs along with an induction of HLA-G expression on T cells. ILT4 on tolerogenic DCs interacts with HLA-G on T cells and HLA-G on DCs interacts with ILT2 on T cells, which drives differentiation of T cells into IL-10 producing Tr1 cells[17]. Monocyte derived human DCs cultured in presence of 1α,25-dihydroxyvitamin D3 (VD3) show a semi-mature phenotype characterized by low levels of MHC Class II and costimulatory molecule expression along with production of IL-10 and impairment of IL-12 production, driving Tr1 differentiation. Moreover, DCs cultured with VD3 upregulate programmed death ligand -1 (PDL-1) upon activation, inhibition of which suppresses Tr1 differentiation[18]. PDL-1 signalling on DCs likely promotes IL-10 production, since triggering PDL-1 on DCs by soluble PD-1 has been shown to suppress DC maturation and promote IL-10 production[19]. Altogether, these studies indicate that VD3 treatment of DCs drives PDL-1 upregulation which acts as an inducer of IL-10 production by DCs, thereby driving Tr1 differentiation. However, it remains to be investigated whether HLA-G and ILT4/ILT2 signalling is involved in VD3 driven induction of Tr1 cells.

Repetitive stimulation of peripheral CD4^+ ^T cells by immature allogeneic DCs can also drive Tr1 generation[20]. T cells cultured under stimulation by immature DCs selectively upregulate cytotoxic T-lymphocyte antigen 4 (CTLA4) and lose their ability to produce IFN-γ, IL-2, IL-4 and subsequently differentiate into Tr1 cells[21]. This is dependent on the ability of immature DCs to secrete IL-10, which is severely diminished as DCs undergo maturation. IL-10 production by pulmonary DCs also appears to be critical for induction of Tr1 induced tolerance. Pulmonary DCs in mice exposed to respiratory antigens undergo maturation but secrete IL-10 which drives induction of Tr1 cells. Moreover, these Tr1 cells can suppress airway responsiveness and their development is dependent on IL-10 production by DCs, since adoptive transfer of DCs from IL-10 deficient mice fail to induce Tr1 mediated tolerance[22]. In a model of food induced anaphylaxis, tolerance induction is mediated by gastrointestinal lamina propria DCs, whereby sugar modified antigens are targeted to C-type lectin receptor SIGNR-1, resulting in preferential production of IL-10 but not IL-6 or IL-12 p70 by DCs, which ends up driving differentiation of naive T cells into Tr1 cells[23].

Several strategies that can induce DC production of IL-10 and prevent maturation have been employed for generation of Tr1 cells. Neuropeptide vasoactive intestinal peptide is released during inflammatory/autoimmune conditions, which induces generation of DCs with a capacity to produce IL-10 and an inability to undergo complete maturation, thereby driving generation of Tr1 cells[24]. Exposure of DCs to cyclo-oxygenaase-2 overexpressing gliomas results in IL-10 production by DCs which also drives Tr1 response, which may be dependent on robust secretion of Prostaglandin E_2 _from the glioma[25]. Additionally, bacterial peptides such as filamentous hemagglutinin from Bortadella pertusis can directly affect DCs by suppressing their ability to produce IL-12 and instead inducing production of IL-10, which drives generation of Tr1 cells[26]. Individuals infected with Plasmodium vivax have elevated levels of Tr1 cells and culture of mononuclear cells from healthy individuals with Plasmodium vivax extracts can drive generation of Tr1 cells, indicating that certain peptides produced from Plasmodium vivax may affect DC function which leads to Tr1 generation[27].

### ICOSL signalling by DCs drives Tr1 differentiation

In addition to IL-10 production by pulmonary DCs, ICOS-ICOSL signalling is also critical for Tr1 induction. Pulmonary DCs upregulate ICOSL upon maturation which is also critical for Tr1 induction since inhibition of ICOSL on pulmonary DCs suppresses Tr1 induction[28]. Plasmacytoid DCs also upregulate ICOS ligand upon maturation, which has been shown to drive differentiation of T cells into Tr1 cells[29].

### TGF-β1 production by DCs drives Tr1 differentiation

TGF-β may also have a role in priming Tr1 differentiation, since addition of neutralizing antibodies against TGF-β to a coculture of CD4^-^CD8^- ^splenic DCs and T cells drastically reduces the production of IL-10 by T cells[30]. CD4^-^CD8^- ^spenic DC subsets secrete elevated levels of TGF-β upon stimulation with lipopolysaccharide (LPS) and subsequently prime differentiation of IL-10 producing Tr1 cells[30].

### Other DC driven signals which drive Tr1 differentiation

Along with TGF-β, CD40 signaling also plays a role in the ability of CD4^-^CD8^- ^DCs to prime Tr1 generation, since CD40 ligation abrogates the ability of CD4^-^CD8^- ^DCs to prime Tr1 cells[31]. Furthermore, TLR9 stimulation via CpG DNA and TLR4 stimulation via LPS can convert these Tr1 inducing DCs into Th1 and Th1/Th17 inducing DCs respectively[23]. Mature pDCs isolated from peripheral blood of rheumatoid arthritis patients express high levels of indolamine-2,3-dioxygenase, which has also been implicated in promoting differentiation of T cells into Tr1 phenotype[32].

## Foxp3^+ ^Regulatory T cells

Foxp3^+ ^regulatory T cells (Foxp3^+ ^Tregs) are critical for maintaining immune tolerance and preventing autoimmune reactions[33]. Foxp3^+ ^Tregs are identified by their expression of CD25 (IL-2 receptor) and Foxp3, a transcription factor critical for Treg differentiation[34]. Foxp3^+ ^Treg population can be divided into the naturally occurring Foxp3^+ ^Treg population (nTreg), generally found in the thymus and the inducible Treg population (iTreg), which is derived in the peripheral tissues from CD4^+^CD25^- ^precursors upon activation in presence of TGF-β[35]. DCs are critical for Treg induction and in this section we offer an insight in the recent advances in our understanding of how DCs can drive nTreg and iTreg differentiation.

## Naturally occurring Foxp3^+ ^Tregs

Natural Foxp3^+ ^Tregs (nTregs) comprise a distinct lineage pathway determined at the double positive (CD4^+^CD8^+^) stage of thymocyte development due in part to co-stimulatory signals initiating Foxp3 expression. nTregs develop in the thymus during thymic development upon recognition of self antigens. Although the role of DCs in thymic selection is documented, the role of DCs in generation of nTregs is highly controversial[36]. Several studies have shown that DCs are dispensable for nTreg generation, whereby antigens specifically expressed in thymic epithelial cells are sufficient to drive differentiation of nTregs [37]. Conversely, there are studies identifying contribution of thymic DCs to generation of nTregs[38]. Thymic DCs include two conventional DC subsets, which are CD8^lo^Sirpα^hi/+ ^and CD8^hi^Sirpα^lo/-^, among which CD8^lo^Sirpα^hi/+ ^DCs have been shown to play a role in inducing nTreg generation in addition to their role in negative selection[39].

### TSLP drives thymic DC mediated nTreg differentiation

Epithelial cells in Hassall's corpuscles in the thymus produce thymic stromal lymphopoetin (TSLP) which acts on thymic DCs by binding to TSLPR and IL-7R alpha complex and drives induction of CD80, CD86[38]. These DCs subsequently prime differentiation of CD4^+^CD8^-^CD25^- ^thymocytes into nTregs, which is dependent on IL-2 and CD28 signaling [40]. Therefore, TSLP activated myeloid DCs in the thymus are likely critical for positive selection of medium to high affinity self reactive thymocytes to develop into nTregs[38]. In addition to myeloid DCs, plasmacytoid DCs (pDCs) residing in the thymus can also induce differentiation of CD69^hi^TCR^hi^CD4^+^CD8^+ ^thymocytes into nTregs and this is dependent on CD40L crosstalk[41]. Thymic pDCs also express TSLP receptor along with IL-7 receptor complex and become responsive to TSLP produced by thymic epithelial cells of Hassall's corpuscles. TSLP activated pDCs can then drive differentiation of nTregs from CD4^+^CD8^-^CD25^- ^thymocytes, which can be inhibited by Th1 and Th2 polarizing chemokines IL-12 and IL-4 respectively[42].

The role of TGF-β in driving nTreg differentiation is highly controversial. Previous studies have shown normal nTreg numbers in TGFβ-R1 deficient mice[35]. However, recently it has been shown that mice with TGFβ-R1 deletion have nTreg deficiency between postnatal day 3 and 5, but subsequently there is a surge in nTreg generation due to increased responsiveness of the cells to IL-2[43]. It remains to be investigated whether thymic DCs produce TGF-β1 which can affect nTreg generation.

## Inducible Foxp3^+ ^Regulatory T cells

**Figure 2:**
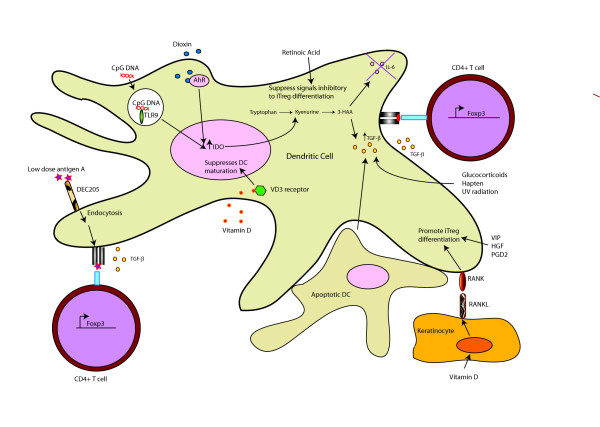
**DCs drive differentiation of foxp3^+ ^inducible regulatory T cells**. **(iTregs)**. DCs secrete TGF-β, which induces foxp3 in naive T cells, driving differentiation of naive T cells into iTregs. Activation of AhR and TLR9 drives induction of IDO, which catalyzes tryptophan metabolism. Tryptophan metabolites promote iTreg generation through induction of TGF-β production and suppression of Th17 inducing cytokine, IL-6. Furthermore, uptake of apoptotic DCs by viable DCs along with exposure to haptens, glucocorticoids and UV radiation also induces TGF-β production, which drives iTreg differentiation. Other signals such as RANK/RANKL signalling by vitamin D treated keratinocytes and treatment of DCs with vasoactive intestinal peptide (VIP), hepatocyte growth factor (HGF) and prostaglandin-D2 (PGD2) also promote iTreg differentiation. Moreover, retinoic acid promotes iTreg differentiation by suppressing cytokines which are inhibitory to iTreg differentiation and targeting of antigen to DEC205 drives iTreg differentiation through a TGF-β dependent mechanism.

Inducible Foxp3^+ ^regulatory T cells (iTregs) are generated in the periphery by DCs and their generation appears to be dependent on IDO, retinoic acid, Vitamin D and TGF-β. iTregs cells play essential roles in immune tolerance and in the control of severe chronic allergic inflammation[44]. Furthermore, since iTregs are induced in the periphery, they also act as barriers in preventing the clearance of microorganisms and tumors, whereby both are known to generate conditions that can drive iTreg differentiation[45,46]. Figure 2 provides an overview of the pathways involved in DC mediated iTreg differentiation.

### Indoleamine 2,3-dioxygenase in DCs drives iTreg differentiation

DC populations expressing indoleamine 2,3-dioxygenase (IDO) can play a critical role in immune tolerance by promoting iTreg induction[47]. IDO catalyzes tryptophan metabolism via the kynurenine pathway and therefore depletes the local environment of tryptophan. Tryptophan catabolism likely plays an important role in suppressing T cell proliferation by arresting T cells in G1 phase of cell cycle[48]. However, recent studies have highlighted an important role of tryptophan catabolites in mediating iTreg induction by exerting their effects directly on DCs. During HIV infection, IDO activity is critical in regulating Treg/Th17 balance with increased IDO levels produced by DCs, associated with a chronic inflammatory state in progressive HIV disease due to a breakdown of mucosal barrier[49]. Certain DC subsets such as pDCs, certain splenic DCs populations such as CD19^+ ^DCs and nasal DCs have been identified to upregulate IDO upon stimulation[50]. Induction of IDO in DCs appears to be dependent on aryl hydrocarbon receptor (AhR), for DCs lacking Ahr fail to upregulate IDO and prime T cell response instead of tolerance induction[51]. Activation of Ahr in mice by 2,3,7,8-Tetrachlorodibenzo-*p*-dioxin(TCDD), commonly referred to as dioxin, for 10 days, results in IDO induction both in the lungs and spleen along with upregulation of Foxp3 in the spleen, which could be suppressed by inhibiting IDO[52].

DCs residing in the nasal lymph nodes play an important role in inducing tolerance to inhaled antigens. Studies have identified selective induction of IDO in non-plasmacytoid DCs in the nasal lymph nodes, which is critical for inducing tolerance, for abrogation of IDO induction results in elimination of tolerance induction towards the inhaled antigen[53]. In a murine model of experimental autoimmune encephalitis, IDO deficient mice show exacerbation of encephalitis, which can be inhibited by treatment with tryptophan metabolite 3-hydroxyanthranilate (3-HAA), generated during IDO mediated tryptophan catabolism. Treatment with 3-HAA drives TGF-β production from DCs and also suppresses IL-6 production, which ends up driving iTreg induction[54]. Kynurenine, the first metabolite of IDO driven tryptophan metabolism activates the AhR on T cells, thereby driving iTreg differentiation[55]. TLR9 ligation drives induction of IDO in pDCs which suppresses IL-6 production, suppressing conversion of naive T cells into Th17 cells and instead promoting iTreg induction[56]. IDO driven kynurenine generation also appears to be important for pDC mediated iTreg differentiation [57]. Human monocyte derived DCs, cultured under low tryptophan conditions, selectively upregulate inhibitory receptors ILT3 and ILT4 and drive iTreg induction[58].

### TGF-β production by DCs drives iTreg differentiation

Skin DCs include langerhans cells (LCs) and dermal DCs, with LCs being frequently associated in maintenance of immune tolerance for acute depletion of LCs has been associated with an enhancement of dermal immune response[59]. Patients with langerhans cell histiocytosis, a condition with uncontrolled proliferation of LCs, show expansion of Foxp3^+ ^Treg populations, indicating a role of LCs in Foxp3^+ ^Treg expansion[60]. Exposure of UVR-exposed skin to haptens results in induction of iTregs, which is not observed upon LC depletion, supporting the role of LCs in inducing iTregs and suppressing immune reaction in the skin[61]. Mice with LC specific TGF-β depletion, show signs of autoimmune disease in the skin and fail to develop LCs, indicating a role for LC derived TGF-β in LC development and also pointing towards a role of LC in maintenance of immune tolerance[62]. TGF-β production by LCs could in fact be a potential mechanism of how LCs can prime differentiation of Tregs. In nickel allergy patients, administration of oral glucocorticoids leads to TGF-β production by LCs, which expands iTregs and results in reduction of clinical symptoms[63].

CD8α^+ ^DCs were initially identified in the mouse spleen with a propensity to drive iTreg induction[64]. A unique CD8^+ ^splenic DC subset, which expresses DEC205, a type I transmembrane protein with multiple C-type lectin domains, has been identified in the mouse spleen, and preferentially drives differentiation of iTregs[65]. CD8^+^DEC205^+ ^DCs can drive iTreg differentiation both *in vitro *and *in vivo *in presence of low dose of the antigen without addition of any exogenous TGF-β. However, iTreg induction mediated by CD8^+^DEC205^+ ^DCs is dependent on TGF-β for addition of TGF-β neutralizing antibody suppresses iTreg differentiation[65]. Furthermore, Polyinosinic:polycytidylic acid (poly I:C) induced maturation of CD8^+^DEC205^+ ^DCs reduces their ability to drive iTregs, pointing towards their role in maintaining peripheral tolerance under steady state conditions. Targeting of small amounts of antigen to DCs by using antigen fused to DEC205 antibody under conditions of suboptimal DC activation has been shown to drive iTreg induction[66]. Another CD8^- ^splenic subset, which expresses DCIR-2, a type II transmenbrane protein with a single external C-type lectin domain, drives iTreg differentiation when exogenous TGF-β is added. However, in absence of exogenous TGF-β, CD8^- ^splenic DCs are better at stimulating nTregs rather than driving iTreg differentiation[65].

Pulmonary DCs mediate inhalational tolerance which occurs during non-inflammatory settings through CCR7 dependent migration of pulmonary DCs to the draining bronchial lymph node[67]. In the absence of inflammatory signal, pulmonary DCs acquire antigens and subsequently acquire a semi mature phenotype characterized by intermediate expression of costimulatory molecules and high levels of MHC II expression, followed by subsequent migration to bronchial lymph nodes, where tolerance is induced[68]. Additionally, local microenvironment in the lung also plays a role in driving iTregs. Pulmonary stromal cells can produce cytokines such as TGF-β which can drive differentiation of pulmonary DC into IL-10 and TGF-β producing DCs which can subsequently drive iTreg differentiation[69].

Uptake of apoptotic DCs by viable DCs suppresses DC maturation and instead induces production of TGF-β1 via the mTOR signalling pathway [70-72]. TGF-β1 producing DCs subsequently interact with naive T cells and drive Foxp3 induction, thereby driving iTreg differentiation.

### RANKL signalling on DCs drives iTreg differentiation

Local environment within skin could also contribute towards maintenance of tolerance by DCs. The interplay between vitamin D and RANKL-RANK signalling in the skin plays a role in inducing an environment which promotes DC induced iTreg induction. The activated metabolite of vitamin D (1,25-dihydroxyvitamin D_3_, VD_3_) exerts actions through its nuclear receptor, the VD_3 _receptor (VDR) [73]. VDR is expressed on immune cells such as DCs and Vitamin D treatment of DC inhibits maturation along with their ability to prime alloreactive T cell response[74]. Keratinocytes in the inflamed skin over express RANKL, which through RANKL-RANK signalling modulates the function of DCs in the epidermis to expand iTregs[75]. Application of topical vitamin D analog, calcipotriol, followed by transcutaneous immunization with a protein agent results in induction of iTregs, primarily due to induction of RANKL on keratinocytes which likely modulates DC function to drive iTreg differentiation[76]. iTreg induction upon topical application of vitamin D is absent in mice lacking vitamin D receptor, indicating vitamin D driven RANKL as the likely mechanism of how vitamin D can mediate skin tolerance by inducing iTregs [76].

### Retinoic acid producing DCs drive iTreg differentiation

Oral intake of protein antigens leads to induction of oral tolerance, which is largely mediated through generation of iTregs in the mesenteric lymph nodes. DCs in the mesenteric lymph nodes express cyclooxygenase-2 (cox-2), which plays a role in iTreg induction. Suppression of cox-2 in mesenteric DCs results in induction of GATA-3 along with IL-4 in T cells and suppresses iTreg induction[77]. In addition to cox-2, mesenteric DCs express high levels of B7-H1 and B7-DC, which are B7 family costimulatory molecules and are also essential for mesenteric DC driven induction of iTregs[78]. The most well studied mechanism of how oral tolerance induces iTregs is through retinoic acid. Retinoic acid (RA) is an active metabolite of vitamin A which regulates multiple cellular processes such as cell death, proliferation and differentiation through the retinoic acid receptors (RAR, including α, β and γ subtypes) and the retinoic × receptors (RXR, also including α, β and γ subtypes). RA has been shown to suppress inflammatory responses in animal models of multiple diseases such as inflammatory bowel disease and experimental autoimmune encephomyelitis. A unique population of DCs, characterized by the expression of alpha E integrin, CD103 has been identified in the gut associated lymphoid tissue (GALT) as well as in the mesentereic lymph node with a specialized function of inducing Tregs and maintaining immune tolerance[79,80]. CD103^+ ^DCs selectively drive iTreg differentiation through RA and TGF-β dependent process, since addition of inhibitors of RA production or TGF-β neutralizing antibody suppresses iTreg induction[79,80]. CD103^+ ^DC derived RA also drives induction of α4β7 integrin and CCR9 on newly generated iTregs, which makes them home to GALT[81]. Furthermore, RA also sustains stability and function of iTregs, even under inflammatory setting which further results in tolerance induction[82]. The mechanism of how RA can drive iTreg induction is not completely understood. Initially, RA was thought to inhibit effects of IL-6 signaling, which promoted iTreg induction rather than induction of Th17 in presence of TGF-β[83]. Later studies indicated that RA suppresses the generation of CD44^hi ^effector memory T cells, which secrete IL-4, IL-21 and IFN-γ, and suppress TGF-β mediated iTreg differentiation[84]. However, RA can also interfere with the effects of inhibitory cytokines on iTreg differentiation and can promote iTreg induction in absence of inhibitory cytokines, which is dependent on RAR-α[85]. Immune deficient mice lacking CD103 fail to suppress T cell mediated colitis upon transfer of Tregs, pointing towards a role of CD103^+ ^DCs in maintaining intestinal immune homoestasis [86]. Curcumin treatment of bone marrow derived DCs drives expression of Aldh1a, an enzyme involved in RA production, which makes DCs behave similarly to mucosal CD103^+ ^DCs and drive RA mediated induction of iTregs[87]. The local intestinal environment also modulates DC function. Intestinal epithelial cells produce TGF-β, retinoic acid which drive a tolerogenic DC phenotype, which can then subsequently drive iTreg differentiation[88,89]. Additionally, lamina propria macrophages also suppress intestinal DC induced Th17 response, which could inadvertently prime iTreg differentiation[90].

CD103^-^CD11b^+ ^DC subset has been identified in the skin and expresses 3 aldehyde dehydrogenases, which catalyze conversion of retinal to RA, giving this subset the unique property of priming iTreg differentiation[91]. Furthermore, treatment of mice with AhR ligand 2-(1'H-indole-3'-carbonyl)-thiazole-4-carboxylic acid methyl ester (ITE) has been shown to result in generation of tolerogenic DCs which promote iTreg differentiation through a RA dependent mechanism[92].

### Other DC derived signals which drive iTreg differentiation

Mast cell derived Prostaglandin-D2 (PGD2) is a mediator of inflammation which promotes infiltration of eosinophils and Th2 cells into the lung during asthma. PGD2 can act through DP1 or DP2 receptor. Studies have shown that treatment of asthmatic mice with DP1 agonist can in fact suppress features of asthma by acting on pulmonary DCs and inducing cAMP dependent protein kinase A activation, which suppresses the ability of DCs to drive Th2 response and instead promotes induction of iTregs[93].

Additionally, treatment of DCs with immunosuppressive peptides has also been shown to drive iTreg differentiation. DCs treated with immunosuppressive neuropeptide, vasoactive intestinal peptide (VIP) drive iTreg differentiation, which is likely mediated by the ability of VIP to suppress DC maturation and proinflammatory cytokine production[94,95]. Moreover, DCs upon treatment with hepatocyte growth factor (HGF) also drive iTreg differentiation, which is abrogated if DCs are treated with antibodies against HGF receptor[96]. However, the mechanisms of how HGF receptor signalling in DC drives iTreg differentiation is not understood.

## Th3 cells

Th3 cells were first identified as a novel population of T cells induced upon induction of peripheral tolerance upon oral delivery of myelin basic protein, which suppressed experimental autoimmune encephalitis in mice[97]. These Th3 cells are class II-restricted T cells with identical αβ TCR as Th1 and Th2 cells. Moreover, they are characterized by production of high levels of TGF-β along with low amounts of IL-4 and IL-10 with no production of IFN-γ or IL-2. The ability of these cells to suppresses EAE is largely TGF-β dependent. Secretion of TGF-β by Th3 cells drives induction of Foxp3 in activated T cells, driving them towards iTreg phenotype[98]. Furthermore, Foxp3 can also be induced in Th3 cells for studies have shown that transient induction of TGF-β1 in T cells during activation in absence of IL-2 can drive generation of Foxp3^+ ^Th3 cells which comprise a distinct Treg phenotype, which is CD25^- ^and can control hyperproliferative T cell response[99].

## Double negative regulatory T cells

TCR^+^CD3^+^CD4^-^CD8^- ^double negative (DN) regulatory T cells inhibit immune response by Fas/FasL destruction of effector cells in an antigen specific fashion[100]. Although, the mechanisms of how DCs can prime differentiation of DN Tregs is not understood, syngeneic DCs have been successfully utilized for expansion of antigen specific DN Tregs[101].

## Conclusion

DCs play a critical role in the induction of tolerance. One of the active mechanisms whereby DCs induce/maintain tolerance is through induction of Tregs. Over the last decade, significant progress has been made in understanding the DC specific signals that can drive induction of Tregs. These findings can potentially be employed to generate tolerogenic DCs which can be used for tolerance induction in hypersensitivity, autoimmunity as well as transplantation.

## List of abbreviations

AhR: Aryl hydrocarbon receptor; cAMP: Cyclic adenosine monophosphate; CCR7: Chemokine (C-C motif) receptor 7; CTLA4: Cytotoxic T-lymphocyte antigen 4; Cox-2: Cyclooxygenase-2; DCs: Dendritic cells; DN: Double negative; GALT: Gut associated lymphoid tissue; HAA: 3-hydroxyanthranilate; HGF: Hepatocyte growth factor; HIV: Human immunodeficiency virus; HLA: Human leukocyte antigen; ICOS: Inducible T cell costimulator; ICOSL: Inducible T cell costimulator ligand; IDO: Indoleamine 2,3-dioxygenase; IFN: Interferon; IL: Interleukin; ILT: Ig like transcript; iTreg: Inducible Foxp3+ regulatory T cells; LCs: Langerhans cells; LPS: Lipopolysaccharide; MHC: Major histocompatibility complex; mTOR: Mammalian target of Rapamycin; nTreg: Naturally occurring Foxp3+ regulatory T cells; OPG: Osteoprotegerin; pDC: Plasmacytoid dendritic cells; PDL-1: Programmed death ligand - 1; PGD2: Prostaglandin D2; Poly I:C: Polyinosinic:polycytidylic acid; RA: Retinoic acid; RANK: Receptor activator of nuclear factor κB; RANKL: Receptor activator of nuclear factor κB ligand; RAR: Retinoic acid receptors; RXR: Retinoic × receptors; STAT: Signal transducer and activator; TCDD: 2,3,7,8-Tetrachlorodibenzo-p-dioxin; TGF: Tumor Growth Factor; TLR: Toll like receptor; Tr1: Type 1 regulatory T cells; Tregs: Regulatory T cells; TSLP: Thymic stromal lymphopoetin; UVR: Ultraviolet radiation; VD3: 1α,25-dihydroxyvitamin D3; VDR: 1α,25-dihydroxyvitamin D3 receptor; VIP: Vasoactive intestinal peptide.

## Competing interests

The authors declare that they have no competing interests.

## Authors' contributions

RK wrote the manuscript and JH revised the manuscript. All authors have read and approved the final manuscript.
